# Comparative Transcriptomic Analyses of Different Jujube Cultivars Reveal the Co-Regulation of Multiple Pathways during Fruit Cracking

**DOI:** 10.3390/genes13010105

**Published:** 2022-01-02

**Authors:** Lu Hou, Meng Li, Chenxing Zhang, Ningwei Liu, Xinru Liu, Wenhao Bo, Xiaoming Pang, Yingyue Li

**Affiliations:** 1 National Engineering Laboratory for Tree Breeding, College of Biological Sciences and Technology, Beijing Forestry University, Beijing 100083, China; houlu822@163.com (L.H.); limeng@bjfu.edu.cn (M.L.); chenxingzhang@bjfu.edu.cn (C.Z.); liuningwei@bjfu.edu.cn (N.L.); Liuxinru377@bjfu.edu.cn (X.L.); bwh9620@gmail.com (W.B.); xmpang@bjfu.edu.cn (X.P.); 2 Key Laboratory of Genetics and Breeding in Forest Trees and Ornamental Plants, Ministry of Education, College of Biological Sciences and Biotechnology, Beijing Forestry University, Beijing 100083, China; 3 The Tree and Ornamental Plant Breeding and Biotechnology Laboratory of National Forestry and Grassland Administration, College of Biological Sciences and Technology, Beijing Forestry University, Beijing 100083, China

**Keywords:** jujube, fruit cracking, transcriptome, differentially expressed genes, functional enrichment analyses, network

## Abstract

Fruit cracking is a common physiological disorder in many fruit species. Jujube (*Ziziphus jujuba* Mill.) is an economically valuable fruit in which fruit cracking seriously affects fruit yield and quality and causes significant economic losses. To elucidate cracking-related molecular mechanisms, the cracking-susceptible cultivars ‘Cuizaohong’ and ‘Jinsixiaozao’ and the cracking-resistant cultivar ‘Muzao’ were selected, and comparative transcriptome analyses of cracking and non-cracking ‘Cuizaohong’ (CC and NC), cracking and non-cracking ‘Jinsixiaozao’ (CJ and NJ), and non-cracking ‘Muzao’ (NM) were conducted. A total of 131 differentially expressed genes (DEGs) were common to the CC vs. NC and CJ vs. NJ comparisons. To avoid passive processes after fruit cracking, we also mainly focused on the 225 gradually downregulated DEGs in the CJ, NJ, and NM samples. The functional annotation of the candidate DEGs revealed that 61 genes related to calcium, the cell wall, the cuticle structure, hormone metabolism, starch/sucrose metabolism, transcription factors, and water transport were highly expressed in cracking fruits. We propose that expression-level changes in these genes might increase the turgor pressure and weaken mechanical properties, ultimately leading to jujube fruit cracking. These results may serve as a rich genetic resource for future investigations on fruit cracking mechanisms in jujube and in other fruit species.

## 1. Introduction

Chinese jujube (*Ziziphus jujuba* Mill.), which belongs to the family Rhamnaceae, is an economically, ecologically, and culturally valuable fruit crop species in China. Jujube fruits are a rich source of energy (i.e., calories) as well as nutritional and medicinal compounds, including carbohydrates, minerals, vitamin C, and cAMP [1]. Jujube is a major fruit tree species with considerable economic benefits for farmers in China. More than eight million tons of jujube fruits were produced in 2016, and jujube fruit cultivation serves as the primary source of income for nearly 20 million people in China [2]. However, fruit cracking has become a serious problem because it adversely affects the appearance of jujube fruits and increases the chances of microbial infections, which lead to significant losses in fruit yield and quality. Regarding jujube production, yield losses due to fruit cracking range from approximately 30% in normal years to more than 90% in severe years [3].

Fruit cracking disorders are common in many plant species, including apple, sweet cherry, pomegranate, grape, tomato, citrus, and lychee [4]. There are many factors that influence fruit cracking, including external factors (orchard management and environmental conditions) and internal factors (fruit size, sugar content, mineral nutrient content, plant hormone balance, exocarp mechanical properties, and exocarp and mesocarp expansion rates) [5]. Cuartero et al. [6] found that fruit cracking was determined by genetic factors as early as 1981. Transcriptome sequencing (RNA-seq) is a powerful technology that can be used to interpret the functional elements of the genome and to reveal the molecular constituents of cells and tissues [7]. Recently, RNA-seq and differential expression analyses have accelerated the elucidation of the molecular basis of fruit cracking. Using RNA-seq technology, Li et al. [8] identified differentially expressed genes (DEGs) that are involved in water transport, gibberellin (GA) metabolism, abscisic acid (ABA) metabolism, calcium (Ca) transport, and cell wall metabolism that mediated fruit cracking in the lychee cultivar ‘Baitangying’. Wang et al. [9] conducted a transcriptome analysis of the pericarps of three kinds of lychee and identified 101 DEGs that are related to pericarp photosynthesis, unsaturated fatty acid (FA) oxidation, cuticle biosynthesis, and pericarp hormone homeostasis. In atemoya, 16,190 upregulated and 1670 downregulated genes that are related to metabolism, phytohormones, and the cell wall were identified during a comparison between split and non-split fruits [10]. The Gene Ontology (GO) terms assigned to the DEGs involved in the response to tomato fruit cracking were mainly ‘hormone metabolic process’, ‘cell wall organization’, ‘oxidoreductase activity’, and ‘catalytic activity’ [11]. Zhu et al. [5] analyzed the transcriptome of the grape cultivar ‘Xiang Fei’ and reported that the genes related to cell wall metabolism and cuticle biosynthesis may play crucial roles in fruit cracking. Wang et al. [12] developed a preliminary model of the molecular events that control fruit cracking in ‘Nuomici’ lychee on the basis of RNA-seq data.

In jujube, various studies have mainly examined the histological, physiological, biochemical, environmental, and cultural factors influencing fruit cracking. By observing water entry in jujube fruit, researchers found that stomatal characteristics and the fruit surface microcracks that are caused by water absorption were the main factors that led to cracking [13]. Li et al. [14] concluded that jujube fruit cracking was correlated with whether wax synthesis is coordinated with fruit enlargement. The concentration of the extracellular Ca that crosslinks adjacent pectin polymers may be an important determinant of fruit cracking after water is absorbed [15]. Ozturk et al. [16] reported that the decreased cracking rates that are associated with GA_3_ treatment may be related to a thickened cuticle layer. To the best of our knowledge, a few studies have investigated the molecular basis of jujube fruit cracking. A recent study detected three SNPs that were significantly associated with the fruit cracking-related traits of jujube germplasm collections by means of association mapping [17]. Using RNA-seq technology, Liu et al. [18] profiled the DEGs between cracked and normal ‘Huping’ jujube fruits. However, they were unable to accurately characterize jujube fruit cracking because the effects of oxidative stress after fruit cracking were unavoidable during their experiments [8]. Although jujube fruit cracking has been investigated for many years, few advances have been made in clarifying the underlying genetic factors.

We have studied jujube breeding for a long time and have specifically focused on fruit cracking and have observed the continuous fruit cracking of 150 jujube cultivars in the core collection for many years. Based on previous research, the cracking-susceptible cultivars ‘Cuizaohong’ and ‘Jinsixiaozao’ and the cracking-resistant cultivar ‘Muzao’ were selected to study the molecular mechanisms that are associated with cracking [17,19]. In this study, we compared the transcriptomes of the half-red fruits from cracking ‘Cuizaohong’ (CC), non-cracking ‘Cuizaohong’ (NC), cracking ‘Jinsixiaozao’ (CJ), non-cracking ‘Jinsixiaozao’ (NJ), and non-cracking ‘Muzao’ (NM) using RNA-seq analysis. Through Venn diagram and trend analyses of the different genotypes, the important DEGs were identified. Functional categorization and co-expression analysis of cracking-related genes were conducted to reveal the various metabolic pathways that are involved in fruit cracking. Our study provides candidate genes for genetically enhancing cracking tolerance to increase fruit quality and yield and also lays the foundation for future studies on the molecular mechanisms underlying fruit cracking in jujube and in other fruit species.

## 2. Materials and Methods

### 2.1. Plant Materials

**Figure 1:**
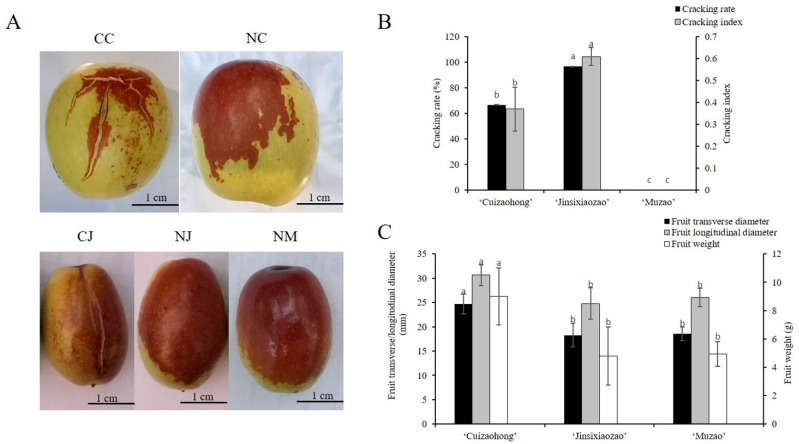
The phenotypes of the jujube at the half-red period (**A**) and evaluation of cracking resistance (**B**) and fruit size (**C**) of three jujube cultivars. CC: cracking ‘Cuizaohong’; NC: non-cracking ‘Cuizaohong’; CJ: cracking ‘Jinsixiaozao’; NJ: non-cracking ‘Jinsixiaozao’; NM: non-cracking ‘Muzao’, the same as below. Error bars represent standard errors from three biological replicates. Different lowercase letters indicate a significant difference between genotypes (*p* < 0.05).

The three jujube cultivars ‘Cuizaohong’, ‘Jinsixiaozao’, and ‘Muzao’ that were used in our experiments were provided by the National Foundation for Improved Cultivars of Chinese Jujube, Cangzhou County, Hebei Province, China. The trees had been subjected to the same orchard management strategy. A total of thirty uniform intact half-red fruits of each jujube cultivar were collected with three biological replicates, in which ten individuals per replicate were used for fruit cracking and fruit size measurements. The half-red fruit from the CC, NC, CJ, NJ, and NM cultivars were collected for RNA-seq, and the cracking and non-cracking fruit samples from the same cultivar were collected from the same tree for each replicate (Figure 1A). The sampling data was 10 September 2017. The collected fruit were divided into three biological replicates, with each containing at least three fruits. Fruits without stones were frozen immediately in liquid nitrogen and were stored at −80 °C for RNA extraction.

### 2.2. Measurements of Fruit Cracking and Fruit Size

The fruit cracking (cracking rate and cracking index) and fruit size (fruit transverse diameter, fruit longitudinal diameter, and fruit weight) of the three jujube cultivars were measured for the three-year period of 2015–2017. Jujube fruit cracking was induced by means of a natural immersion method, and the cracking rate was measured at 48 h, as described by Du et al. [20]. The cracking index was calculated according to the grading system for jujube fruit cracking [21]. The transverse and longitudinal diameters (maximum value) of fruits were measured using a vernier caliper, and the fruit weight was measured with the help of a digital electric balance. 

### 2.3. RNA Extraction and Sequencing

Total RNA was isolated using TRIzol (Invitrogen, Carlsbad, CA, USA) in accordance with the manufacturer’s instructions. The RNA concentration was quantified using a NanoDrop 2000 (Thermo Fisher Scientific, Wilmington, DE, USA), and RNA integrity was evaluated using an RNA Nano 6000 Assay Kit on the Agilent Bioanalyzer 2100 system (Agilent Technologies, Palo Alto, CA, USA). A total amount of 1 μg RNA per sample was used as input material for the RNA sample preparations. Sequencing libraries were generated using the NEB Next Ultra small RNA Sample Library Prep Kit for Illumina (NEB, Ipswich, MA, USA) following the manufacturer’s recommendations, and index codes were added to attribute sequences to each sample. The clustering of the index-coded samples was performed on a cBot Cluster Generation System using a TruSeq PE Cluster Kit v4-cBot-HS (Illumina, San Diego, CA, USA) in accordance with the manufacturer’s instructions. After cluster generation, the library preparations were sequenced on an Illumina HiSeq Xten platform, and paired-end reads were generated. The isolation of the mRNA, fragment interruption, cDNA synthesis, adapter addition, PCR amplification, and RNA-seq were performed by Biomarker Technologies Co., Ltd. (Beijing, China).

### 2.4. Sequence Data Analysis

Raw data (raw reads) in the fastq format were first processed using in-house perl scripts. In this step, clean data (clean reads) were obtained by removing reads containing adapters and poly-Ns as well as low quality reads from the raw data. For the clean data, the percentages of the bases whose Phred values were more than 20 (Q20) and 30 (Q30) [22], the content of guanine and cytosine for the library in one tissue (GC content), and the frequency of reads with the same sequences (sequence duplication level) were calculated. The remaining high-quality clean sequencing reads were mapped onto the ‘Dongzao’ reference genome sequence (version 1.1) [23] using Tophat2 software [24].

### 2.5. Differential Expression Analysis

The jujube gene expression levels were estimated using fragments per kilobase of transcript per million fragments mapped (FPKM) [25]. Differential expression analyses of two samples were performed using edgeR software [26]. A *p*-value < 0.05 and an absolute value of log_2_ fold change (FC) ≥ 1 were used as thresholds to determine the significant differences in gene expression levels.

The DEGs were clustered using the Short Time-series Expression Miner (STEM) software (http://www.sb.cs.cmu.edu/stem). The DEGs with similar expression patterns were included in the same profile. The profiles with *p* < 0.05 were identified as significantly enriched modules.

### 2.6. Functional Annotation, Enrichment Analysis and Transcription Factor Identification

Gene function was annotated based on the following databases: the NCBI nonredundant protein database (Nr), the Clusters of Orthologous Groups database (COG), Swiss Institute of Bioinformatics databases (Swiss-Prot), and the evolutionary genealogy of genes: Nonsupervised Orthologous Groups database (eggNOG) using BLAST (E value ≤ 1 × 10^−5^). Gene Ontology functional enrichment and the Kyoto Encyclopedia of Genes and Genomes (KEGG) pathway analyses were carried out using the GOseq R packages [27] and KOBAS software [28]. DEGs were significantly enriched in GO terms, and KEGG pathways were determined at *p*-values ≤ 0.05. Software iTAK was used to identify transcription factors (TFs) [29].

### 2.7. Co-Expression Analysis

The co-expression between DEGs was analyzed using SPSS (version 19) by calculating the Spearman’s correlation coefficients of their FPKM values. Then, the co-expression of these DEGs was visualized using Cytoscape [30].

### 2.8. Validation by qRT-PCR Analysis

Total RNA was isolated from fruit tissue as described above. The first-strand cDNA was synthesized from 500 ng total RNA using a FastQuant RT Kit (with gDNase) (Tiangen, Beijing, China) in accordance with the manufacturer’s instructions. The specific primers were designed using Beacon Designer 7.9 for qRT-PCR analysis. The *ZjACT* gene was used as an internal control (housekeeping gene) [31]. All of the primer sequences that were used for qRT-PCR are provided in Table S1. qRT-PCR was carried out on an ABI 7500 Fast Real-Time PCR System (Applied Biosystems, USA) using SYBR green (Aidlab Biotechnology, Beijing, China) in a 20 μL reaction mixture containing 1 μL of diluted cDNA, 400 nM of each primer, and 10 μL 2 × SYBR Green PCR Master Mix. The PCR reaction protocol was 94 °C for 3 min, 40 cycles of 94 °C for 20 s, 55 °C for 20 s, and 72 °C for 30 s. Relative gene expression levels were determined using the 2^−ΔΔCt^ method.

### 2.9. Statistical Analysis

SPSS 19.0 and EXCEL 2013 were used for statistical analysis. Analysis of statistical significance was performed according to one-way analysis of variance (ANOVA) with Duncan. Each experiment was repeated three times independently, and *p* < 0.05 was considered to represent significance.

## 3. Results

### 3.1. Analysis of Fruit Characteristics in Three Jujube Cultivars

The cracking resistance of three jujube cultivars were measured for three continuous years. Two cultivars, ‘Cuizaohong’ and ‘Jinsixiaozao’, exhibited a high cracking rate and cracking index, but no ‘Muzao’ fruit were cracked (Figure 1B). Based on the cracking rate and index, ‘Cuizaohong’ and ‘Jinsixiaozao’ were cracking-susceptible cultivars, and ‘Muzao’ was a strong cracking-resistant cultivar. In terms of fruit size, there was no significant difference in terms of the fruit transverse diameter, fruit longitudinal diameter, and fruit weight between ‘Jinsixiaozao’ and ‘Muzao’, which demonstrated contrasting crack resistance (Figure 1C), which indicated that these two cultivars are ideal materials for investigating the fruit cracking mechanisms in jujube [19]. 

### 3.2. Transcriptome Sequencing Results

Three independent replicates of each fruit sample (CC, NC, CJ, NJ, and NM) were subjected to RNA-seq analyses to identify the potential molecular mechanisms that were responsible for jujube fruit cracking. A total of 26.36, 26.32, 29.15, 27.45, and 28.83 million raw reads were obtained from the CC, NC, CJ, NJ, and NM transcriptome libraries, respectively (Table 1). After quality control, more than 84.21% clean reads (the percentages of Q30 and GC being 86.48–91.50% and 44.26–46.12%, respectively) were obtained and were used for downstream analyses (Table S2). The alignment results showed that most of the clean reads (70.30–73.59%) from the 15 libraries could be mapped to the reference genome (Table S3). On average, approximately 32.13 (62.23%), 32.59 (59.93%), and 35.34 (62.54%) million reads were uniquely mapped on the reference genome using Tophat2 for ‘Cuizaohong’, ‘Jinsixiaozao’, and ‘Muzao’, respectively. From the saturation curve, even the genes with low expression levels (FPKM < 1) were saturated (Figure S1). This indicated that the number of obtained reads was sufficient to cover most of the expressed genes. The gene coverage analysis showed no bias in the curves. The results showed that the reads were evenly distributed among the genes in the reference genome (Figure S2). Overall, the data indicated that our Illumina sequencing was of high quality and could be used for further analyses.

### 3.3. Analysis of DEGs among Different Jujube Cultivars

**Figure 2:**
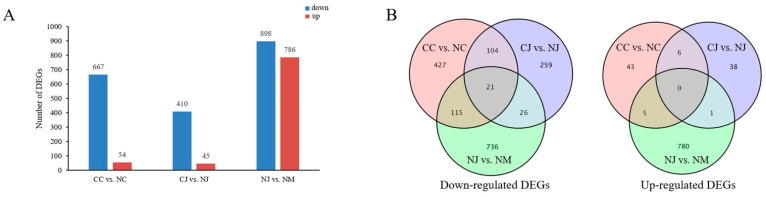
Analysis of DEGs in three jujube cultivars. (**A**) Numbers of DEGs in CC vs. NC, CJ vs. NJ, and NJ vs. NM. (**B**) Venn diagrams of DEGs numbers and distributions among three comparisons.

Three pairwise comparative analyses (CC vs. NC, CJ vs. NJ, and NJ vs. NM) of the DEGs systematically explored the potential molecular mechanisms of fruit cracking within and between jujube cultivars. In total, 721 DEGs (54 upregulated and 667 downregulated) were detected in CC vs. NC, 455 DEGs (45 upregulated and 410 downregulated) were detected in CJ vs. NJ, and 1684 DEGs (786 upregulated and 898 downregulated) were detected in NJ vs. NM (Figure 2A). A Venn diagram indicated the DEG distribution among the three comparisons (Figure 2B). A total of 21 downregulated genes overlapped in CC vs. NC, CJ vs. NJ, and NJ vs. NM. An analysis of the DEGs was performed between the two cracking-susceptible jujube cultivars (‘Cuizaohong’ and ‘Jinsixiaozao’), and 131 DEGs showed similar expression patterns in the cracking and non-cracking fruits, reflecting the common cracking-related genes in both cultivars. When these two cultivars were exposed to fruit cracking, the number of downregulated genes (125 DEGs) was greater than the number of upregulated genes (six DEGs), and ‘Cuizaohong’ had more DEGs than ‘Jinsixiaozao’, indicating more complex cracking response pathways in ‘Cuizaohong’.

In ‘Cuizaohong’, the log_2_FC of the DEGs ranged from −11.27 to 2.59, while in ‘Jinsixiaozao’, this parameter fluctuated between −4.68 and 4.38 (Table S4). In ‘Cuizaohong’, *gene5475* was the most downregulated gene (log_2_FC = −11.27). It was annotated as encoding an unknown protein. The *gene30138* showed the greatest increase in expression (log_2_FC = 2.59) and was also annotated as encoding an unknown protein. In ‘Jinsixiaozao’, *gene5490* exhibited the greatest expression level (log_2_FC = −4.68) and encoded linoleate 13S-lipoxygenase 2-1, chloroplastic. The expression levels of *gene28904* displayed the most dramatic repression (log_2_FC = 4.38) and was also annotated as encoding an unknown protein. Because the functions of the most up- and downregulated genes in each library are unknown, further analyses to identify their functions are needed.

### 3.4. Functional Annotation of DEGs

**Figure 3:**
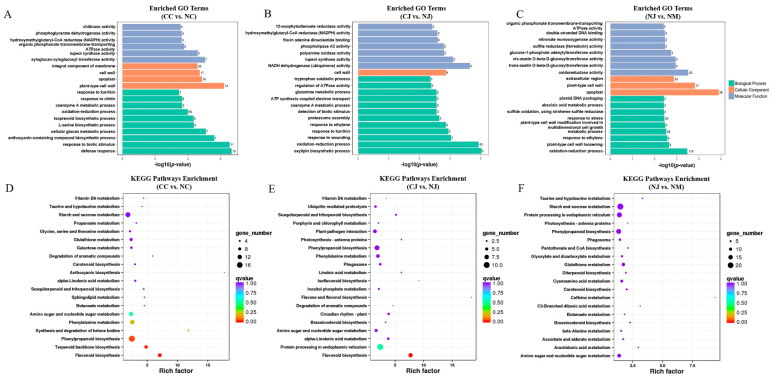
The enrichment analysis of the DEGs using GO and KEGG pathways. (**A**–**C**) GO classifications of DEGs in CC vs. NC, CJ vs. NJ, and NJ vs. NM, respectively. The x-axis indicates −log10 (*p*-value), number of DEGs, and the y-axis indicates the top 20 enriched GO terms. The numbers on the bar graph show the number of genes enriched in each term. (**D**–**F**) The top 20 enriched KEGG pathways of the DEGs in CC vs. NC, CJ vs. NJ, and NJ vs. NM, respectively. The rich factor is the ratio of DEGs and the total number of detected genes in the pathway. The bubble size represents the number of DEGs detected in each KEGG pathway. The color of the bubble represents the *q*-value.

To determine the functions of the DEGs, their sequences were used as queries to screen six databases. In total, 710 (98.47%), 444 (97.58%), and 1621 (96.26%) DEGs in the CC vs. NC, CJ vs. NJ, and NJ vs. NM comparisons, respectively, matched sequences in at least one database (Table S5). A GO analysis was performed to classify the DEGs according to their functions. Among the DEGs, 434 (37.94%), 246 (52.09%), and 930 (41.50%) in the CC vs. NC, CJ vs. NJ, and NJ vs. NM comparisons, respectively, were assigned GO terms (Table S5). The most common biological process GO term among the DEGs that were detected in the three comparisons was the ‘oxidation-reduction process’. In the molecular function category, ‘lupeol synthase activity’ and ‘hydroxymethylglutaryl-CoA reductase (NADPH) activity’ were significantly enriched among the DEGs that were detected in the comparisons of cracking and non-cracking fruits (CC vs. NC and CJ vs. NJ). However, regarding the NJ vs. NM comparison, the most enriched molecular function GO term was ‘oxidoreductase activity’. For all of the comparisons, the significantly enriched cellular component GO terms were ‘cell wall’ and ‘plant-type cell wall’ (Figure 3A–C). 

To identify the biological pathways that are activated in the selected jujube fruits, we mapped the annotated sequences to the reference pathways in the KEGG database. In this analysis, 80, 62, and 104 significantly enriched functional categories were identified for the DEGs in the CC vs. NC, CJ vs. NJ, and NJ vs. NM comparisons, respectively (Table S6). Two pathways (‘Amino sugar and nucleotide sugar metabolism’ and ‘phenylpropanoid biosynthesis’) were common among the top 20 KEGG pathways for the DEGs detected in the three comparisons (Figure 3D–F). For the two cracking-susceptible jujube cultivars, ‘flavonoid biosynthesis’ was significantly enriched among the DEGs that were revealed by the comparison of the cracking and non-cracking fruits (CC vs. NC and CJ vs. NJ, Figure 3D,E). However, in the NJ vs. NM comparison, most of the DEGs were associated with ‘starch and sucrose metabolism’ (Figure 3F).

### 3.5. Venn Diagram and Trend Analyses

**Figure 4:**
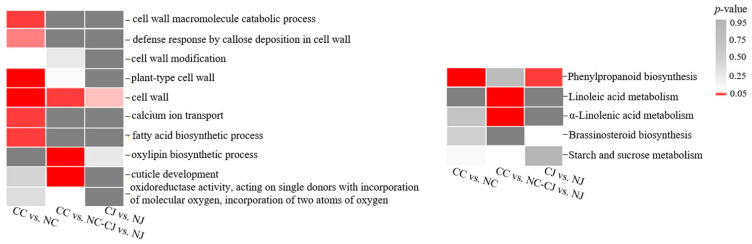
Results of the GO (left) and KEGG (right) enrichment analysis of the DEGs in two cracking-susceptible cultivars. The *p*-value was used to indicate the significance of the most represented GO and KEGG terms. Significant *p*-values are indicated in red, whereas nonsignificant *p*-values are indicated in dark gray.

The functional annotation of the DEGs in the two cracking-susceptible cultivars revealed common genes that were related to unsaturated FA oxidation and the cuticle structure (‘oxylipin biosynthetic process’; ‘cuticle development’; ‘oxidoreductase activity, acting on single donors with incorporation of molecular oxygen, incorporation of two atoms of oxygen’; ‘linoleic acid metabolism’; and ‘α-linolenic acid metabolism’) (Figure 4). Additionally, the common genes that were annotated with ‘cell wall’ and ‘starch and sucrose metabolism’ were also identified in both cracking-susceptible cultivars. In ‘Cuizaohong’, the DEGs were associated with the cell wall (‘cell wall macromolecule catabolic process’, ‘defense response by callose deposition in cell wall’, ‘cell wall modification’, ‘plant-type cell wall’, and ‘cell wall’), the cuticle structure (‘FA biosynthetic process’ and ‘phenylpropanoid biosynthesis’), and the Ca ion (‘Ca ion transport’). In ‘Jinsixiaozao’, the DEGs were annotated with ‘cell wall’ and ‘brassinosteroid (BR) biosynthesis.’ We mainly focused on the common DEGs that were involved in cracking-related metabolic pathways, including the genes encoding pectinesterase 40 (*PME40*, *gene28523*), xyloglucan endotransglucosylase/hydrolase 2 (*XTH2*, *gene14330* and *gene19193*), lupeol synthase (*LUS*, *gene14606* and *gene14733*), UDP-glucuronate 4-epimerase 6 (*GAE6*, *gene14375*), and 9-cis-epoxycarotenoid dioxygenase NCED1, chloroplastic (*NCED1*, *gene30271*) (Table S7). 

**Figure 5:**
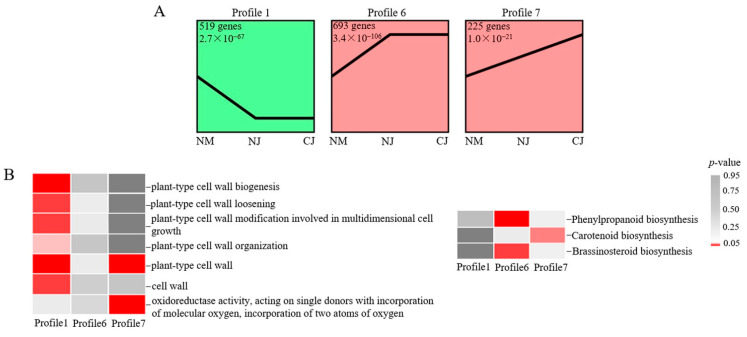
Gene expression patterns in NM, NJ, and CJ samples. (**A**) Gene expression patterns in three samples predicted with STEM software. The number of genes and *p*-values for each pattern are indicated in the frame. (**B**) Results of the GO (left) and KEGG (right) enrichment analysis of important processes in three samples. The significance of the most represented terms is indicated by a *p*-value. Significant *p*-values are indicated in red, whereas nonsignificant *p*-values are indicated in dark gray.

To avoid oxidative stress and microbial invasion after fruit cracking, we performed a trend analysis of the DEGs (2013 genes) in the NM, NJ, and CJ samples. The DEGs for the three samples were clustered into eight profiles (Table S8). Profiles 1, 6, and 7 were significantly represented in the two genotypes (Figure 5A). Genes related to the cell wall were enriched in Profiles 1 and 7. Moreover, the genes that were associated with the cuticle structure (‘phenylpropanoid biosynthesis’ and ‘oxidoreductase activity, acting on single donors with incorporation of molecular oxygen, incorporation of two atoms of oxygen’) and hormone metabolism (‘BR biosynthesis’ and ‘carotenoid biosynthesis’) were included in Profiles 6 and 7 (Figure 5B). The expression of the genes in Profiles 1 and 6 did not differ in NJ and CJ. We mainly focused on the Profile 7 DEGs with expression levels that were gradually downregulated in the CJ, NJ, and NM samples (highest and lowest expression levels in CJ and NM, respectively). Genes encoding EXORDIUM (*EXO*, *gene10454* and *gene28757*), epidermis-specific secreted glycoprotein EP1 (*EP1*, *gene23768*), peroxidase (*PER*, *gene7752*), gibberellin 2-beta-dioxygenase (*GA2OX1*, *gene643*), naringenin, 2-oxoglutarate 3-dioxygenase (*AN3*, *gene16247*), and *NCED1* (*gene1854* and *gene30271*) were grouped in Profile 7 (Table S8). 

### 3.6. Identification of Differentially Expressed Transcription Factors

**Figure 6:**
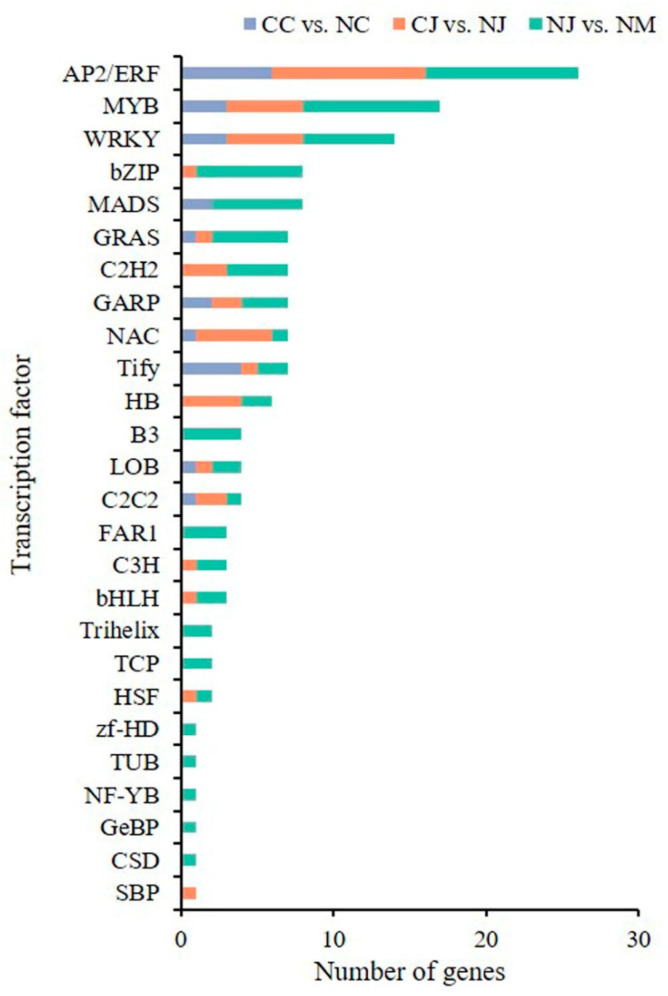
Transcription factor families of the DEGs in CC vs. NC, CJ vs. NJ, and NJ vs. NM.

To explore the roles of TFs in fruit cracking, we analyzed the differential expression of the TFs in CC vs. NC, CJ vs. NJ, and NJ vs. NM. A total of 124 TFs from 26 families, such as *AP2/ERF* (18), *MYB* (13), *WRKY* (10), and *bZIP* (eight), were differentially expressed in all three groups (Figure S3). Among these TFs, 24 were in CC vs. NC, 44 were in CJ vs. NJ, and 79 were in NJ vs. NM (Figure 6). The most abundant TFs were *AP2/ERF* family members in each comparison.

### 3.7. Co-Expression Analysis of Cracking-Related Genes

**Figure 7:**
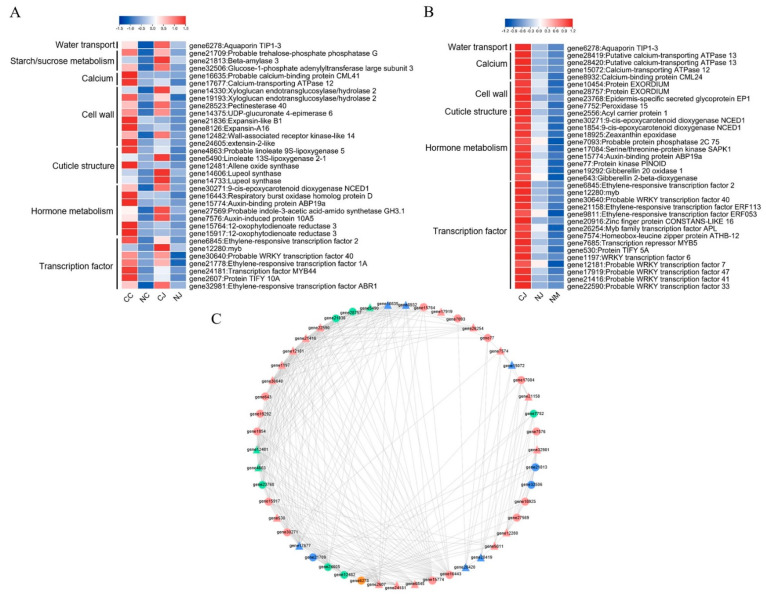
Co-expression of cracking-related genes. Heatmaps of the expression of candidate genes related to fruit cracking in two cracking-susceptible cultivars (**A**) and in cracking-susceptible and -resistant cultivars (**B**). (**C**) Network representation of cracking-related genes (orange circles: water transport, green circles: cell wall, blue circles: starch and sucrose metabolism, pink circles: hormone metabolism, green triangles: cuticle structure, blue triangles: calcium, pink triangles: transcription factor). Nodes represent genes, and edges represent relationships between any two genes. Edges represent positive correlations as determined by a Spearman correlation coefficient > 0.7.

To explore the DEG functions related to the regulation of jujube fruit cracking, we functionally annotated candidate DEGs, including 131 genes common to the CC vs. NC and CJ vs. NJ comparisons as well as 225 gradually downregulated genes in the CJ, NJ, and NM samples. The DEGs in the Venn diagram and trend analyses were divided into seven groups (Ca, cell wall, cuticle structure, hormone metabolism, starch/sucrose metabolism, TF, and water transport) (Figure 7A,B). Regarding the CC, NC, CJ, and NJ samples, the expression levels of 33 cracking-related DEGs (one water transport gene, eight cell wall metabolism genes, three starch and sucrose metabolism genes, five cuticle structure genes, two Ca transport genes, two ABA metabolism genes, three indoleacetic acid (IAA) metabolism genes, two jasmonic acid (JA) metabolism genes, and seven TFs) were upregulated in the cracking fruits of the two cracking-susceptible jujube cultivars (Figure 7A). In the CJ, NJ, and NM samples, the expression levels of 34 cracking-related DEGs (one water transport gene, four cell wall metabolism genes, one cuticle structure gene, four Ca transport genes, five ABA metabolism genes, two IAA metabolism genes, two GA metabolism genes, and fifteen TFs) were gradually downregulated (Figure 7B). Additionally, one water transport gene (*gene6278*), one ABA metabolism gene (*gene30271*), one IAA metabolism gene (*gene15774*), and three TFs (*gene6845*, *gene12280*, and *gene30640*) were common to the two analyses. 

The expression levels of the genes related to Ca, cell wall, cuticle structure, hormone metabolism, starch/sucrose metabolism, TF, and water transport in jujube underwent a network analysis to examine the relationship between these processes. The expression levels of one water transport gene, nine cell wall metabolism genes, three starch and sucrose metabolism genes, five cuticle structure genes, six Ca transport genes, fourteen hormone metabolism genes, and seventeen TFs were highly correlated (Figure 7B). The cell wall metabolism-related, hormone metabolism-related, and TF expression levels were positively correlated with the expression of the analyzed cracking-related genes. Moreover, the expression levels of the water transport-related and cuticle structure-related genes were highly positively correlated with the expression of the starch/sucrose metabolism-related and Ca-related genes, respectively.

### 3.8. qRT-PCR Validation

**Figure 8:**
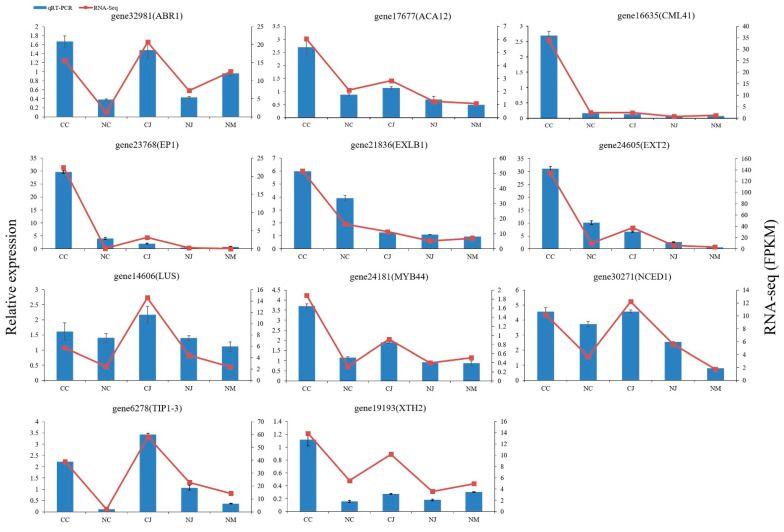
Comparison of RNA-seq results and qRT-PCR analysis of gene expression levels. Error bars represent standard errors from three biological replicates.

To validate the transcriptome data, we selected 11 cracking-related genes and evaluated their expression profiles using qRT-PCR. The gene-specific primers used in the qRT-PCR analysis are listed in Table S1. Figure 8 illustrates the expression levels of the DEGs as determined by qRT-PCR and RNA-seq. The qRT-PCR results indicated that the expression profiles of the 11 DEGs were consistent with the corresponding FPKM values derived from the RNA-seq analysis. These genes exhibited a similar expression pattern when both methods were used. The similar results that were obtained from the qRT-PCR and RNA-seq analyses indicated that the RNA-seq data were reproducible and reliable. 

## 4. Discussion

Fruit cracking is a serious disorder that negatively affects fruit marketability. Our limited knowledge of the molecular characteristics of jujube has made it difficult to recommend appropriate methods to prevent fruit cracking. In the present study, we hypothesized that jujube fruit cracking is due to an imbalance in the turgor pressure and exocarp mechanical strength, which is regulated by a set of genes (Figure S4).

Fruit cracking is the result of excessive water uptake, which causes the exocarp to burst [4]. In the current study, one DEG (*gene6278*) involved in water transport was more highly expressed in the cracking fruits than it was in the non-cracking fruits, and its expression was gradually downregulated in the CJ, NJ, and NM samples (Figure 7A,B). This DEG encodes the aquaporin TIP1-3, which facilitates the transport of water and small neutral solutes across cell membranes [32]. In lychee, an aquaporin gene is commonly more highly expressed in cracking fruits than it is in non-cracking fruits [8]. We speculated that *TIP1-3* may be the key gene that controls jujube fruit cracking by regulating water transport.

The osmotic potential of a fruit is another factor that is correlated with cracking. Sugar is an important component of fruits because of its role in fruit growth and development. An increase in the total soluble sugar content in maturing fruits leads to a decrease in the tissue osmotic potential, which results in increased water absorption and growth as well as a greater likelihood of cracking [33]. Li et al. [34] reported that the total soluble sugar content is higher in cracking-susceptible cultivars than it is in cracking-resistant cultivars. Regarding the comparisons of the DEGs between cracking and non-cracking fruits, ‘starch and sucrose metabolism’ was revealed as an enriched KEGG pathway among the common DEGs. Additionally, the expression of starch/sucrose metabolism-related genes (*gene21709*, *gene21813*, and *gene32506*) was upregulated in cracking fruits (Figure 7A). This finding is consistent with the results of an earlier study by Wang et al. [35], in which starch/sucrose metabolism-related genes were highly expressed in the cracking fruits of cracking-susceptible cultivars. In the present study, the expression of starch/sucrose metabolism-related genes was positively associated with the expression of water transport-related genes. The transcripts for the genes involved in starch/sucrose metabolism were highly abundant in the cracking fruits of the cracking-susceptible cultivar, which may result in a decrease in the tissue osmotic potential. Notably, the *TIP* transcript levels were also high in the cracking fruits, which may lead to an increase in the amount of water entering fruits during heavy rainfall, thereby accelerating fruit cracking.

The mineral content is correlated with fruit cracking, and a preharvest spray application of mineral salts can restrict the uptake of water by fruits [33,36]. Calcium is critical for maintaining the structural integrity and firmness of fruit cell walls because it decreases cell wall permeability, leading to restricted water uptake [37]. The GO term ‘Ca ion transport’ was significantly enriched among the DEGs that were detected in the CC vs. NC comparison (Figure 4). Two Ca transport-related genes (*gene16635* and *gene17677*) were more highly expressed in the cracking fruits than they were in the non-cracking fruits. Moreover, four Ca transport-related genes (*gene15072*, *gene28419*, *gene28420*, and *gene8932*) had gradually downregulated expression levels in the CJ, NJ, and NM samples (Figure 7B). These DEGs encode the Ca-binding protein CML (*CML*) or Ca-transporting ATPase (*ACA*), which are crucial for plant growth and development as well as for responses to various external stimuli [38,39]. The *CML* and *ACA* expression levels are reportedly downregulated in the cracked lychee pericarp [8]. According to Yang et al. [40], in cracking ‘Lingwu Long-jujube’ fruits, Ca^2+^ precipitates are distributed at low levels in the exocarp cells, which is in contrast to the relatively large amounts of Ca^2+^ precipitates in the mesocarp cell walls, cell wall edges, and intercellular space. The discrepancy between our results and those of an earlier investigation on lychee may be associated with the differences in the examined plant materials. In our study, the exocarp and mesocarp were sampled together for sequencing analyses. Accordingly, future studies will need to analyze the exocarp and mesocarp separately.

The exocarp developmental stages and mechanical properties, which are closely related to cell wall metabolism, affect the occurrence of fruit cracking [41]. In our study, several cell wall metabolism-related GO terms, including ‘cell wall’ and ‘plant-type cell wall’, were assigned to the DEGs that were detected in three comparisons. The Venn diagram and trend analyses confirmed that the metabolic activities of the cell wall were enriched during fruit cracking. According to the DEG annotations, 12 cell wall metabolism-related genes might be involved in jujube fruit cracking, of which two *XTH* genes (*gene14330* and *gene19193*), two *expansin* genes (*EXP*, *gene21836* and *gene8126*), and one *PME* gene (*gene28523*) were more highly expressed in the cracking fruits than in they were in the non-cracking fruits (Figure 7A,B). Similar results were reported for atemoya, in which the expression levels of most *PME*, *EXP*, and *XTH* genes are higher in cracking fruits than they are in non-cracking fruits [10]. The suppressed expression of a *polygalacturonase* (*SlPG*) and *SlEXP1* in ripening tomato fruits decreases cell wall disassembly, which leads to increased cracking resistance [42]. Thus, these cell wall-related genes contribute to cell wall disassembly and decrease the elasticity of the exocarp.

The structure and function of the cuticle, which is an important part of the fruit exocarp, have important effects on the mechanisms underlying fruit cracking [43]. The plant cuticle comprises a complex mixture of aliphatic compounds, which are composed of lignin and highly unsaturated FAs [44]. Lignin is an abundant phenylpropanoid polymer [45], and α-linolenic acid is a major unsaturated FA [46]. In the present study, ‘phenylpropanoid biosynthesis’ was one of the top 20 KEGG pathways among the DEGs detected in the CC vs. NC, CJ vs. NJ, and NJ vs. NM comparisons (Figure 3). Regarding the common DEGs in two comparisons of cracking and non-cracking fruits and the gradually downregulated DEGs in jujube cultivars that are susceptible and resistant to fruit cracking, the functional annotations revealed that these DEGs are related to unsaturated FA oxidation and many processes affecting the cuticle structure (Figure 4). The high expression levels of a *lipoxygenase* (*LOX*) in the lychee cultivar ‘Baitangying’ suggest that the cuticle of this cracking-susceptible cultivar might have relatively few unsaturated FAs [9]. In our study, two *LOX* genes (*gene4863* and *gene5490*) were highly expressed in cracking jujube fruits (Figure 7A), implying that they may positively affect fruit cracking-related networks. Hence, we speculate that the susceptibility of jujube cultivars to fruit cracking is related to the changes in the oxidation of unsaturated FAs and the cuticle structure.

Hormonal homeostasis is an important factor that influences fruit cracking [32]. The accumulation of IAA and ABA in the aril is necessary for the growth and development of the aril, but high IAA or ABA contents can induce fruit cracking in lychee [47]. Auxin regulates cell size, stimulates carbohydrate translocation to the fruit, and increases cell wall elasticity [48]. Applications of ABA increase cracking because this treatment increases water movement into and promotes the enlargement of the fruit [42,49]. In this study, the KEGG functional analysis of Profile 7 (gradually downregulated profile) revealed a significant enrichment in the ABA-related ‘carotenoid biosynthesis’ pathways (Figure 5B). An analysis of the data generated in the current study identified one IAA metabolism-related gene (*gene15774*) and one ABA metabolism-related gene (*gene30271*) with important roles during the fruit cracking process and in cultivars that differ in terms of fruit cracking resistance (Figure 7A,B). The *gene15774* encodes an auxin-binding protein that probably mediates the rapid effects of IAA on cell elongation [50]. The protein encoded by *NCED1* (*gene30271*) catalyzes key ABA biosynthesis steps, resulting in an increase in fruit firmness and an extended shelf-life [51]. Abscisic acid affects cell wall catabolism in ripening tomato fruits by downregulating the expression of major catabolic genes [51]. Our co-expression analysis detected a strong correlation between hormone metabolism and other cracking-related metabolic activities. Consistent with the findings of previous studies on atemoya [10] and lychee [12,47], we observed that the upregulated expression of ABA-related and IAA-related DEGs in cracking jujube fruits can lead to increased ABA and IAA levels in fruits, which might promote fruit cracking directly or indirectly by upregulating the expression of specific TFs, such as *MYB*, *WRKY*, and *NAC*, to increase growth-related gene expression.

Transcription factors play critical roles in responding to various types of abiotic stress, including water stress [52]. In the current study, we determined that the genes encoding TFs from several families, including the *WRKY* (*gene30640*, *gene1197*, *gene12181*, *gene17919*, *gene21416*, and *gene22590*), *MYB* (*gene12280*, *gene24181*, and *gene7685*), and *ERF* (*gene6845*, *gene21778*, *gene32981*, *gene21158*, and *gene9811*) families were more highly expressed in cracking fruits than they were in non-cracking fruits. Moreover, the expression levels of *gene6845* (*ERF*), *gene12280* (*MYB*), and *gene30640* (*WRKY*) were gradually downregulated in the CJ, NJ, and NM samples (Figure 7A,B). Liao et al. [53] identified *ClERF4* as a candidate gene that was responsible for rind hardness, which is associated with watermelon fruit cracking resistance. The overexpression of the grape *VvWRKY11* gene in *Arabidopsis thaliana* can adjust the osmotic potential to increase the resistance to cold conditions [54]. In another study involving *A. thaliana*, the expression of the sheepgrass *LcMYB4* gene positively regulated the accumulation of soluble sugars in transgenic plants [55]. Transcription factors regulate plant growth and development by modulating the transcription of specific target genes [12]. In our study, we revealed a positive correlation between TFs and fruit cracking-related processes. However, precisely how these TFs are involved in jujube fruit cracking remains to be determined.

## 5. Conclusions

We conducted comprehensive RNA-seq analyses of five jujube fruit types, namely CC, NC, CJ, NJ, and NM. A total of 131 DEGs were common to the CC vs. NC and CJ vs. NJ comparisons, and 225 DEGs were gradually downregulated in the CJ, NJ, and NM samples. Functional enrichment analyses suggested that DEGs were related primarily to cell wall metabolism, cuticle structure, and hormone metabolism, which highlight the importance of the metabolic pathway that is involved in fruit cracking. Among the two categories (131 and 225) of DEGs, 33 and 34 genes were closely associated with fruit cracking, respectively. Six genes (*TIP1-3*, *NCED1*, *ABP19A*, *ERF2*, *MYB*, and *WRKY40*) were common to the Venn diagram and trend analyses. The upregulation of the genes associated with starch/sucrose metabolism and water transport might increase the expanding pressure, whereas the genes related to cell wall metabolism and cuticle structure might decrease the exocarp mechanical strength, thereby making jujube fruit susceptible to cracking. The TFs and genes associated with hormones, such as auxin and ABA, play critical roles in the regulation of fruit cracking via the above-named pathways. Our findings may help to elucidate the molecular mechanisms underlying fruit cracking.

## Figures and Tables

**Table 1:** Statistical results of transcriptome sequencing data and mapping rate.

Sample	Raw Reads	Clean Reads	Clean Bases	GC (%)	Q30 (%)	Total Mapped Reads (%)	Uniquely Mapped Reads (%)
CC	26,363,434	25,818,100	7,722,697,490	44.42	91.42	73.16	64.03
NC	26,315,272	25,807,498	7,725,295,650	44.86	86.92	70.40	60.43
CJ	29,154,642	28,593,357	8,556,528,121	44.48	86.91	70.66	62.15
NJ	27,450,994	25,637,345	7,667,051,071	45.45	86.92	71.72	57.72
NM	28,834,319	28,252,252	8,438,681,007	44.51	87.27	71.11	62.54

## Data Availability

The raw data of the RNA-Seq have been submitted to NCBI Sequence Read Archive (SRA) under BioProject accession number PRJNA541230.

## Supplementary Materials

The following are available online at https://www.mdpi.com/article/10.3390/genes13010105/s1, Figure S1: The saturation curve of different ripening stages. Figure S2: The Coverage of sequencing. Figure S3: Distribution of differentially expressed transcription factors in gene families. Figure S4: Hypothesized mechanisms of fruit cracking in jujube. Table S1: Primer used for qRT-PCR. Table S2: Summary of sequence assembly after Illumina sequencing. Table S3: Clean reads mapped to the reference genome. Table S4: Complete lists of differentially expressed genes in three groups based on pairwise comparisons. Table S5: Annotation summaries of DEGs. Table S6: KEGG pathways of the DEGs in CC vs. NC, CJ vs. NJ, and NJ vs. NM. Table S7: DEGs involved in important processes during fruit cracking. Table S8: Details regarding the DEGs used for the trend analysis in the NM, NJ, and CJ samples.
