# Supplementary material for: Comparative Transcriptomic Analyses of Different Jujube Cultivars Reveal the Co-Regulation of Multiple Pathways during Fruit Cracking
Source: Genes (Basel). 2022 Jan 2;13(1):105. doi: 10.3390/genes13010105 (PMC8775106; doi:10.3390/genes13010105)
Supplement: Supplementary file 1 [file genes-13-00105-s001.zip › Figure S2.pdf]

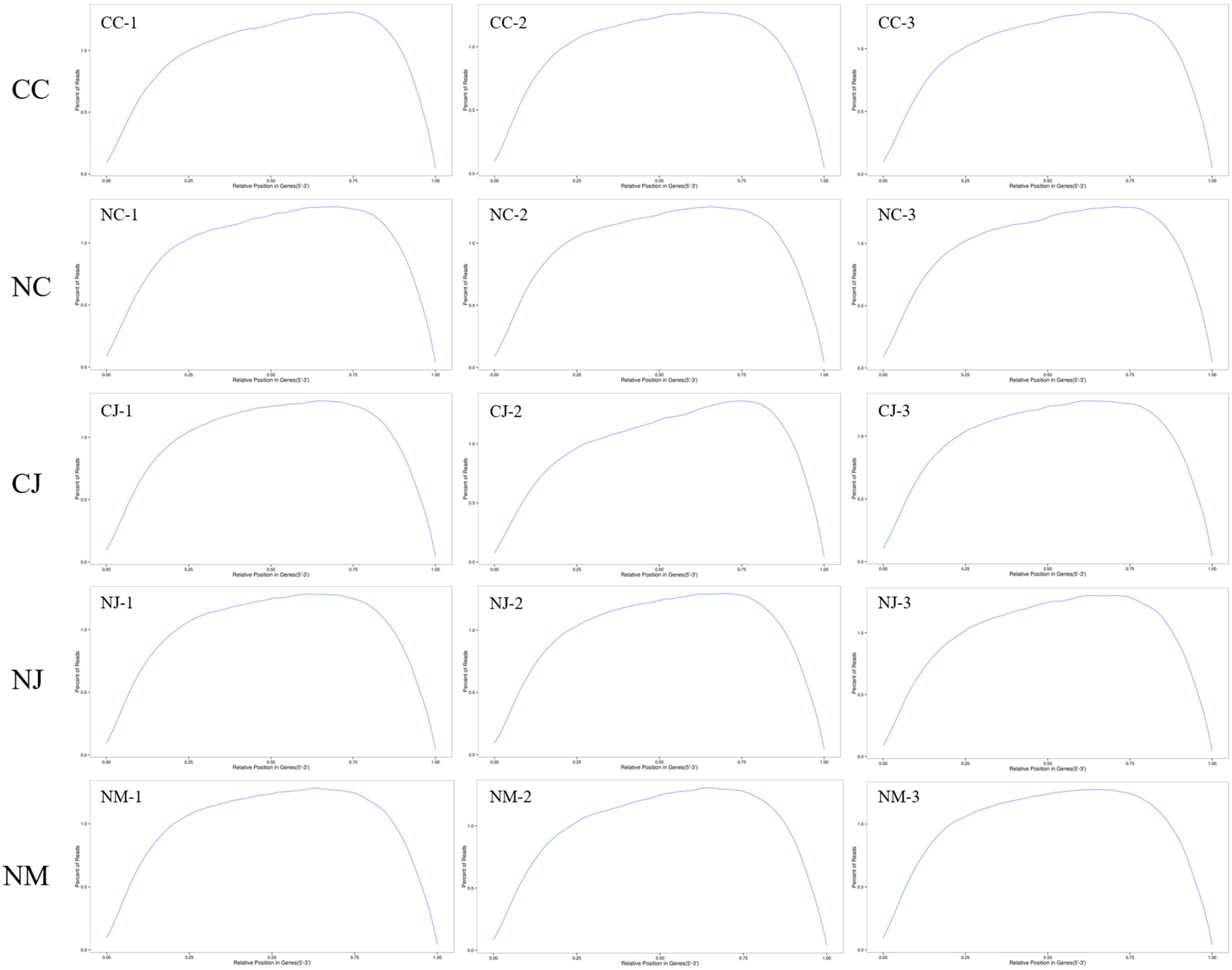

Figure S2. The Coverage of sequencing. The X axis represents the relative position in genes body (from 5’ to 3’). The Y axis represents the percent of reads.
