# Supplementary figures and images for: Comparative Transcriptomic Analyses of Different Jujube Cultivars Reveal the Co-Regulation of Multiple Pathways during Fruit Cracking
Source: Genes (Basel). 2022 Jan 2;13(1):105. doi: 10.3390/genes13010105 (PMC8775106; doi:10.3390/genes13010105)

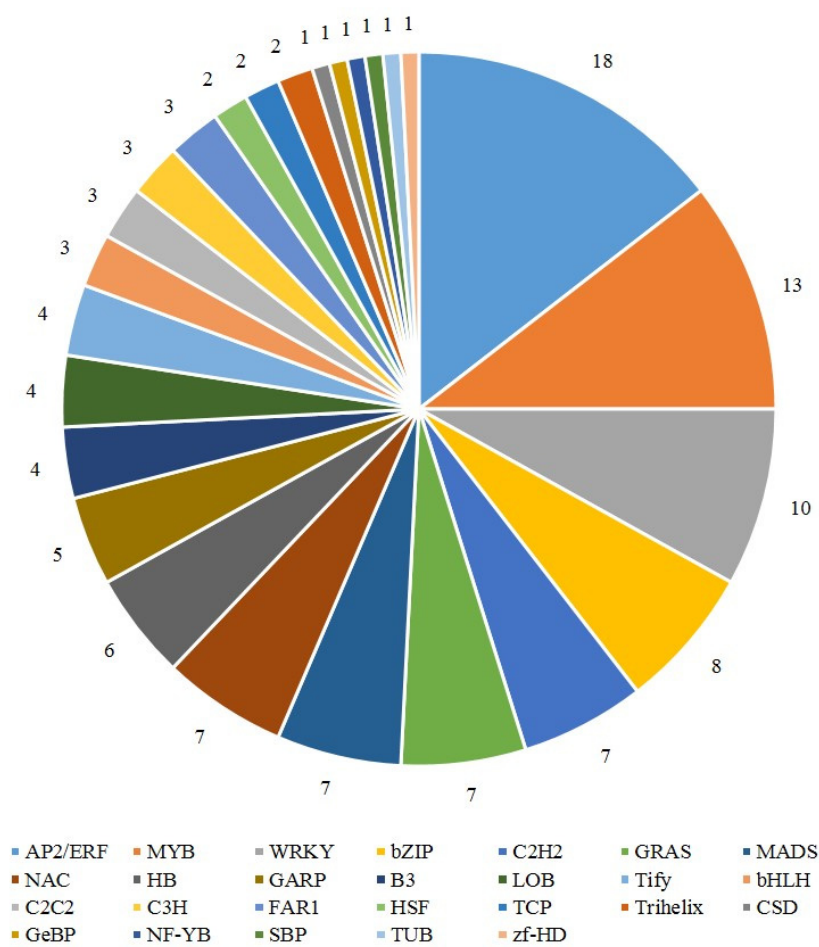

**Figure S3.** Distribution of differentially expressed transcription factors in gene families.

Supplement: Supplementary file 1 [file genes-13-00105-s001.zip › Figure S3.pdf]

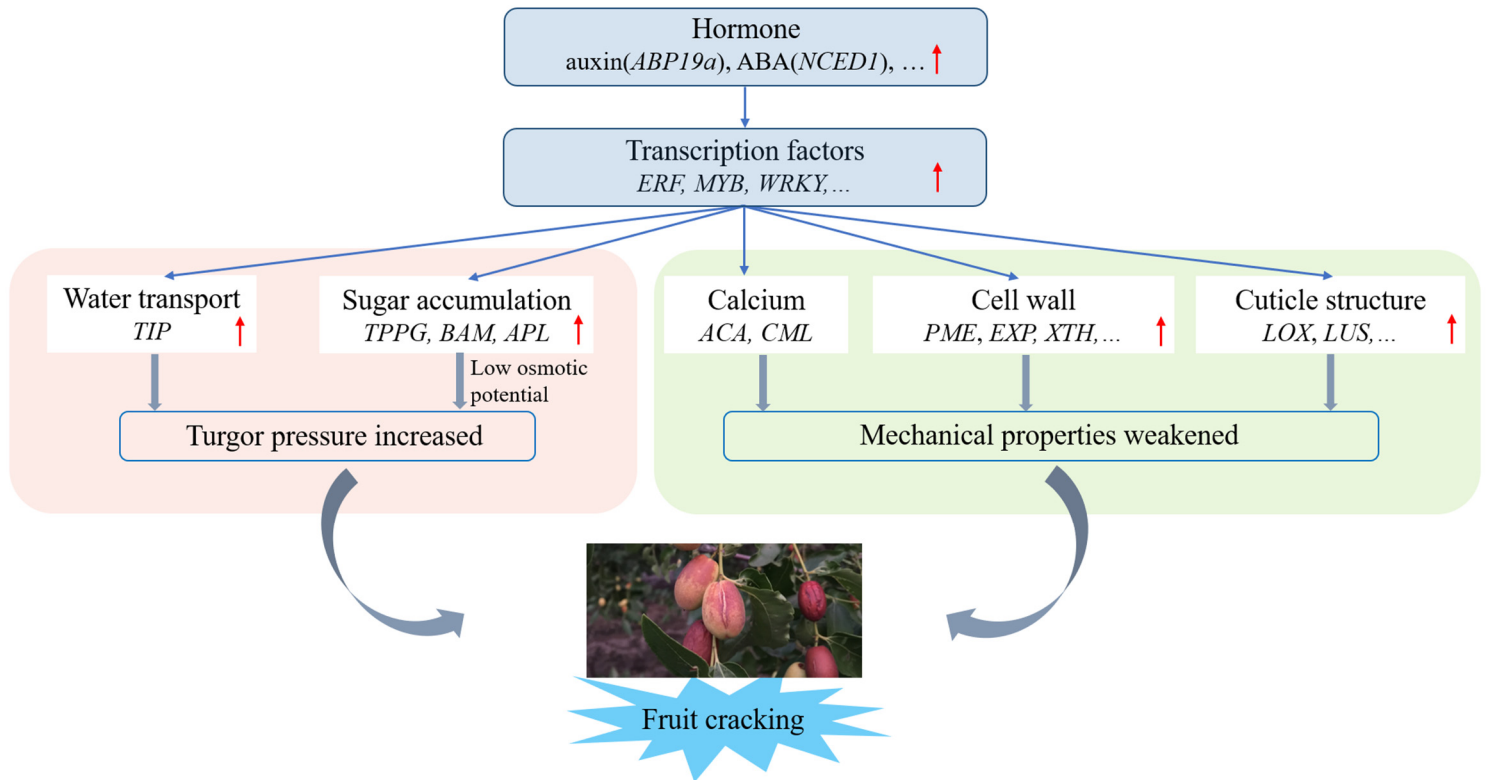

**Figure S4.** Hypothesized mechanisms of fruit cracking in jujube.

Supplement: Supplementary file 1 [file genes-13-00105-s001.zip › Figure S4.pdf]
